# Supplementary figures and images for: Proteome-scale prediction of molecular mechanisms underlying dominant genetic diseases
Source: PLoS One. 2024 Aug 22;19(8):e0307312. doi: 10.1371/journal.pone.0307312 (PMC11341024; doi:10.1371/journal.pone.0307312)

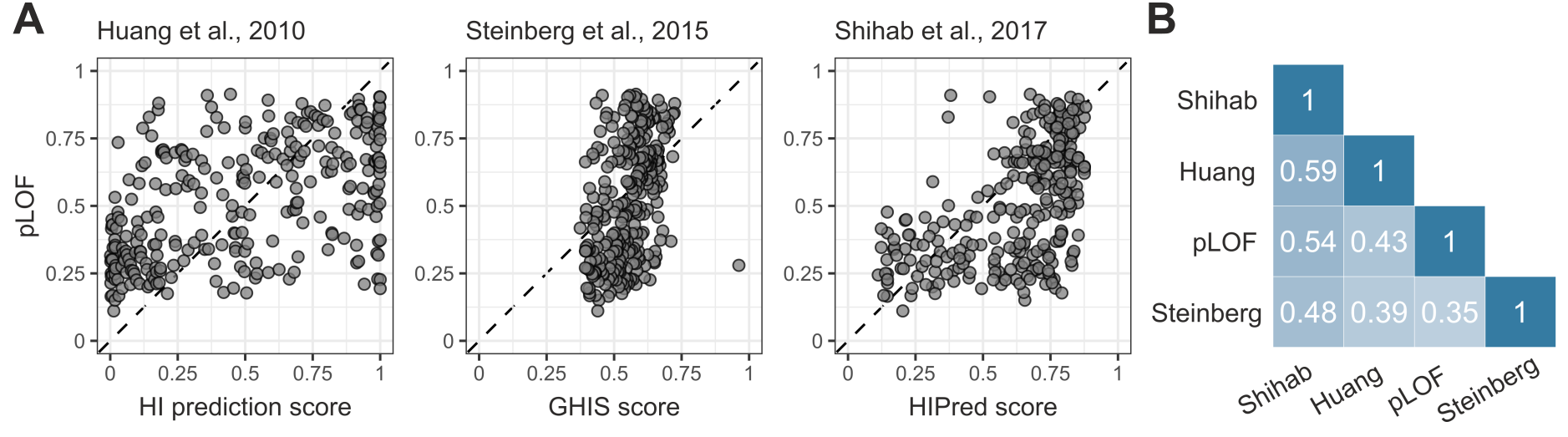

Supplement: S1 Fig — (A) Scatter plots of haploinsufficiency scores from the indicated studies vs pLOF, assessed on the LOF vs non-LOF test set. pLOF has the highest agreement with the Shihab et al. predictions (Pearson’s r = 0.54, p = 4.5×10^-24^) and the lowest with those of Steinberg et al. (r = 0.35, p = 6.9×10^-19^). (B) Pearson correlation triangle showing mutually shared values of all four metrics on human genes (n = 15,046). Although considerable variation exists even among haploinsufficiency predictors, low correlation with pLOF is expected, because haploinsufficiency predictors were not exclusively trained on dominant genes and their negative class comprised of haplo-sufficient genes of mostly no disease relevance. By contrast, pLOF values from the LOF vs non-LOF model were obtained by training the model to recognise haploinsufficient genes against a background of strictly dominant genes with molecular mechanisms other than simple LOF. (TIF) [file pone.0307312.s001.tif]

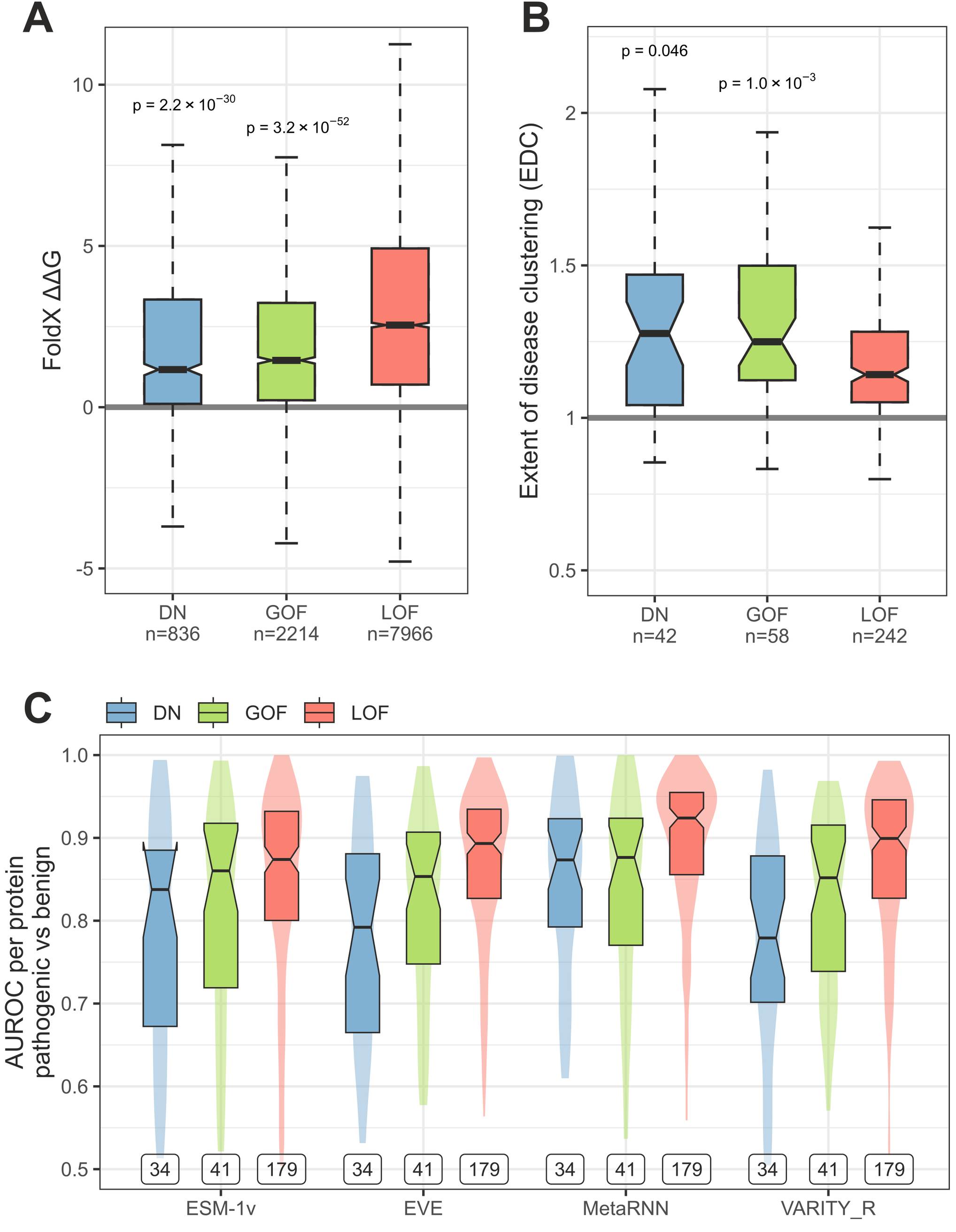

Supplement: S2 Fig — (A) FoldX-predicted ΔΔG of pathogenic missense mutations. Numbers below classes denote the number of mutations. Holm-Bonferroni corrected p-values above DN and GOF boxes are relative to the LOF group and were determined by one-sided Wilcoxon rank-sum test. Sample sizes indicate the number of variants. (B) Class probabilities of the analysis set vs EDC. Sample sizes indicate the number of proteins in each class. Holm-Bonferroni corrected p-values above DN and GOF boxes are relative to the LOF group and were determined by one-sided Wilcoxon rank-sum test. (C) Aggregated AUROC analysis of pathogenic vs benign variants in predicted molecular mechanism classes. Labels indicate the number of proteins in each class. Boxes denote data within 25th and 75th percentiles, the middle line represents the median and the notch contains the 95% confidence interval of the median. Violins show area-normalized distributions. (TIF) [file pone.0307312.s002.tif]
