# Supplementary material for: Proteome-scale prediction of molecular mechanisms underlying dominant genetic diseases
Source: PLoS One. 2024 Aug 22;19(8):e0307312. doi: 10.1371/journal.pone.0307312 (PMC11341024; doi:10.1371/journal.pone.0307312)
Supplement: Attachment — Submitted filename: response_to_reviewers.docx [file pone.0307312.s006.docx]

**Reviewer #1**: The study entitled “*Proteome-scale prediction of molecular mechanisms underlying dominant genetic diseases*” is a reflecting good comprehensive work. However, improvement in language and clarity in sentences are needed. This work is acceptable with minor correction.

Comments:
As per OMIM, there are thousands of “dominant genetic diseases” grouped into autosomal and sex chromosome. Is this article addressing all as one category? What about recessive genetic diseases?

The study necessarily addresses dominant genes as one category. Our goal was to predict the most likely molecular mechanism that would be expected from a gene once dominant disease-associated mutations are identified in them. To this end, we collected reported mechanisms strictly in autosomal dominant genes. These genes may be haploinsufficient (i.e. dominant loss-of-function) or the mutated allele can give rise to a dominant-negative or an assembly-mediated gain-of-function effect. The majority of recessive mutations have a loss-of-function effect, with only a few examples of other mechanisms, *e.g.*, recessive gain-of-function known to date. We have added a sentence in the introduction to bring this information to the reader’s attention: “*Nearly all recessive disorders are associated with LOF, with only a few rare cases of alternative mechanisms identified to date [1]*”.

Author mentioned dominant-negative but ignored dominant-positive mutations.

The reviewer is correct that we did not create a separate category for the dominant-positive effect; there are two reasons for this. First, many dominant-positive effects are likely to have been grouped together with gain-of-function mechanisms in our data, because they essentially represent the assembly-mediated gain-of-function effect [1]. Second, the experimental challenges in precisely characterising molecular mechanisms mean that dominant-positive effects are often not distinctly reported in the literature, preventing their classification as a separate category.

Please enlist the interpretable features were used in the modeling study.

All gene-level features used to train the models, along with their detailed descriptions, are available on the Open Science Framework repository (URL: https://osf.io/9gx5n; see the “features” tab in table_S2.xlsx). Additionally, with the resubmission of this manuscript to PLOS One, we have uploaded all referenced tables to be included in the supplementary material.

**Reviewer #2**: The manuscript entitled "Proteome-scale prediction of molecular mechanisms underlying Dominant Genetic Diseases" presents a comprehensive approach to predicting the molecular mechanisms underlying dominant genetic disorders. In this manuscript, the authors introduce a tripartite statistical model consisting of support vector machine binary classifiers trained to predict whether human protein-coding genes are associated with dominant-negative (DN), gain-of-function (GOF), or loss-of-function (LOF) molecular disease mechanisms. The study estimates the utility of these predictions by examining biologically and clinically important properties known to be associated with the mechanisms. The manuscript is well-written and provides valuable insights into predicting disease mechanisms at a proteome scale. The manuscript is acceptable for publication after minor revision.

Minor Comments
1. In introduction section It would be better to include a brief overview of existing computational approaches for predicting disease mechanisms and how the proposed tripartite statistical model improves upon or complements these methods.

To our knowledge, no gene-level molecular mechanism predictor has been developed thus far. In the original manuscript, under the **Proteome-scale molecular mechanism prediction** section, we included a paragraph and a supplemental figure (**Figure S1**) comparing our model to haploinsufficiency predictors. Our model is likely to be more specific than these predictors, as its component models were trained solely on dominant disease genes to tell apart alternative molecular mechanisms from that of LOF and vice versa.

To highlight this distinction and complement the analysis in the main text, we have added the following sentence to the **Introduction**: “*Given the limitations of current methods that primarily focus on LOF mechanisms, such as haploinsufficiency predictors [49–51], we aimed to develop a method for more directly differentiating between dominant molecular mechanisms.*”

We hope this revision addresses the reviewer’s concern.

2. Please include method layout diagram in the methodology section

We appreciate the reviewer’s suggestion. We have added a workflow diagram (**Figure 1** in the new manuscript) that illustrates the methodology and model validation.

3. Please improve the image quality of Figure 3

We have now submitted TIF files in full resolution to the journal.

4. The inclusion of future directions and potential areas for further research would enrich the manuscript.

We have added a new paragraph to the **Conclusion** section to incorporate future directions for molecular disease mechanism prediction.

**References** (numbering follows the manuscript’s)

1.	Backwell L, Marsh JA. Diverse Molecular Mechanisms Underlying Pathogenic Protein Mutations: Beyond the Loss-of-Function Paradigm. Annual review of genomics and human genetics. 2022;23. doi:10.1146/ANNUREV-GENOM-111221-103208

49.	Izumi K, Nakato R, Zhang Z, Edmondson AC, Noon S, Dulik MC, et al. Germline gain-of-function mutations in AFF4 cause a developmental syndrome functionally linking the super elongation complex and cohesin. Nat Genet. 2015;47: 338–344. doi:10.1038/ng.3229

50.	Seidman JG, Seidman C. Transcription factor haploinsufficiency: when half a loaf is not enough. J Clin Invest. 2002;109: 451–455. doi:10.1172/JCI15043

51.	Kim J, Jiang H, Park S, Eltorai AEM, Stewart FR, Yoon H, et al. Haploinsufficiency of Human APOE Reduces Amyloid Deposition in a Mouse Model of Amyloid-β Amyloidosis. J Neurosci. 2011;31: 18007–18012. doi:10.1523/JNEUROSCI.3773-11.2011
